# Variability in pulmonary vein electrophysiology and fibrosis determines arrhythmia susceptibility and dynamics

**DOI:** 10.1371/journal.pcbi.1006166

**Published:** 2018-05-24

**Authors:** Caroline H. Roney, Jason D. Bayer, Hubert Cochet, Marianna Meo, Rémi Dubois, Pierre Jaïs, Edward J. Vigmond

**Affiliations:** 1 IHU Liryc, Electrophysiology and Heart Modeling Institute, foundation Bordeaux Université, F-33600 Pessac- Bordeaux, France; 2 Division of Imaging Sciences and Biomedical Engineering, King’s College London, London, United Kingdom; 3 Univ. Bordeaux, IMB UMR 5251, CNRS, F-33400 Talence, France; 4 Hôpital Cardiologique du Haut-L’évêque, Université de Bordeaux, LIRYC Institute: IHU LIRYC ANR-10-IAHU-04 and Equipex MUSIC ANR-11-EQPX-0030, Bordeaux, France; Stanford University, UNITED STATES

## Abstract

Success rates for catheter ablation of persistent atrial fibrillation patients are currently low; however, there is a subset of patients for whom electrical isolation of the pulmonary veins alone is a successful treatment strategy. It is difficult to identify these patients because there are a multitude of factors affecting arrhythmia susceptibility and maintenance, and the individual contributions of these factors are difficult to determine clinically. We hypothesised that the combination of pulmonary vein (PV) electrophysiology and atrial body fibrosis determine driver location and effectiveness of pulmonary vein isolation (PVI). We used bilayer biatrial computer models based on patient geometries to investigate the effects of PV properties and atrial fibrosis on arrhythmia inducibility, maintenance mechanisms, and the outcome of PVI. Short PV action potential duration (APD) increased arrhythmia susceptibility, while longer PV APD was found to be protective. Arrhythmia inducibility increased with slower conduction velocity (CV) at the LA/PV junction, but not for cases with homogeneous CV changes or slower CV at the distal PV. Phase singularity (PS) density in the PV region for cases with PV fibrosis was increased. Arrhythmia dynamics depend on both PV properties and fibrosis distribution, varying from meandering rotors to PV reentry (in cases with baseline or long APD), to stable rotors at regions of high fibrosis density. Measurement of fibrosis and PV properties may indicate patient specific susceptibility to AF initiation and maintenance. PV PS density before PVI was higher for cases in which AF terminated or converted to a macroreentry; thus, high PV PS density may indicate likelihood of PVI success.

## Author summary

Atrial fibrillation is the most commonly encountered cardiac arrhythmia, affecting a significant portion of the population. Currently, ablation is the most effective treatment but success rates are less than optimal, being 70% one-year post-treatment. There is a large effort to find better ablation strategies to permanently cure the condition. Pulmonary vein isolation by ablation is more or less the standard of care, but many questions remain since pulmonary vein ectopy by itself does not explain all of the clinical successes or failures. We used computer simulations to investigate how electrophysiological properties of the pulmonary veins can affect rotor formation and maintenance in patients suffering from atrial fibrillation. We used complex, biophysical representations of cellular electrophysiology in highly detailed geometries constructed from patient scans. We heterogeneously varied electrophysiological and structural properties to see their effects on rotor initiation and maintenance. Our study suggests a metric for indicating the likelihood of success of pulmonary vein isolation. Thus either measuring this clinically, or running patient-specific simulations to estimate this metric may suggest whether ablation in addition to pulmonary vein isolation should be performed. Our study provides motivation for a retrospective clinical study or experimental study into this metric.

## Introduction

Success rates for catheter ablation of persistent atrial fibrillation (AF) patients are currently low; however, there is a subset of patients for whom pulmonary vein isolation (PVI) alone is a successful treatment strategy [1]. PVI ablation may work by preventing triggered beats from entering the left atrial body, or by converting rotors or functional reentry around the left atrial/pulmonary vein (LA/PV) junction to anatomical reentry around a larger circuit, potentially converting AF to a simpler tachycardia [2]. It is difficult to predict whether PVI represents a sufficient treatment strategy for a given patient with persistent AF [1], and it is unclear what to do for the majority of patients for whom it is not effective.

Patients with AF exhibit distinct properties in effective refractory period (ERP) and conduction velocity (CV) in the PVs. For example, paroxysmal AF patients have shorter ERP and longer conduction delays compared to control patients [3]. AF patients show a number of other differences to control patients: PVs are larger [4]; PV fibrosis is increased; and fiber direction may be more disorganised, particularly at the PV ostium [5]. There are also differences within patient groups; for example, patients for whom persistent AF is likely to terminate after PVI have a larger ERP gradient compared to those who require further ablation [1, 3].

Electrical driver location changes as AF progresses; drivers (rotors or focal sources) are typically located close to the PVs in early AF, but are also located elsewhere in the atria with longer AF duration [6]. Atrial fibrosis is a major factor associated with AF and modifies conduction. However, there is conflicting evidence on the relationship between fibrosis distribution and driver location [7, 8].

It is difficult to clinically separate the individual effects of these factors on arrhythmia susceptibility and maintenance. We hypothesise that the combination of PV properties and atrial body fibrosis determines driver location and, thus, the likely effectiveness of PVI. In this study, we tested this hypothesis by using computational modelling to gain mechanistic insight into the individual contribution of PV ERP, CV, fiber direction, fibrosis and anatomy on arrhythmia susceptibility and dynamics. We incorporated data on APD (action potential duration, as a surrogate for ERP) and CV for the PVs to determine mechanisms underlying arrhythmia susceptibility, by testing inducibility from PV ectopic beats. We also predicted driver location, and PVI outcome.

## Materials and methods

### Bilayer model

All simulations were performed using the CARPentry simulator (available at https://carp.medunigraz.at/carputils/). We used a previously published bi-atrial bilayer model [9], which consists of resistively coupled endocardial and epicardial surfaces. This model incorporates detailed atrial structure and includes transmural heterogeneity at a similar computational cost to surface models. We chose to use a bilayer model rather than a volumetric model incorporating thickness for this study because of the large numbers of parameters investigated, which was feasible with the reduced computational cost of the bilayer model.

As previously described, the bilayer model was constructed from computed tomography scans of a patient with paroxysmal AF, which were segmented and meshed to create a finite element mesh suitable for electrophysiology simulations. Fiber information was included in the model using a semi-automatic rule based method that matches histological descriptions of atrial fiber orientation [10]. The left atrium of the bilayer model consists of linearly coupled endocardial and epicardial layers, while the right atrium is an epicardial layer, with endocardial atrial structures including the pectinate muscles and crista terminalis. The left and right atrium of the model are electrically connected through three pathways: Bachmann’s bundle, the coronary sinus and the fossa ovalis. Tissue conductivities were tuned to human activation mapping data from Lemery et al. [9, 11].

The Courtemanche-Ramirez-Nattel human atrial ionic model was used with changes representing electrical remodelling during persistent AF [12], together with a doubling of sodium conductance to produce realistic action potential upstroke velocities [9], and a decrease in I_K1_ by 20% to match clinical restitution data [13]. Regional heterogeneity in repolarisation was included by modifying ionic conductances of the cellular model, as described in Bayer et al. [14], which follows Aslanidi et al. and Seemann et al. [15, 16]. Parameters for the baseline PV model were taken from Krueger et al. [17].

**Fig 1:**
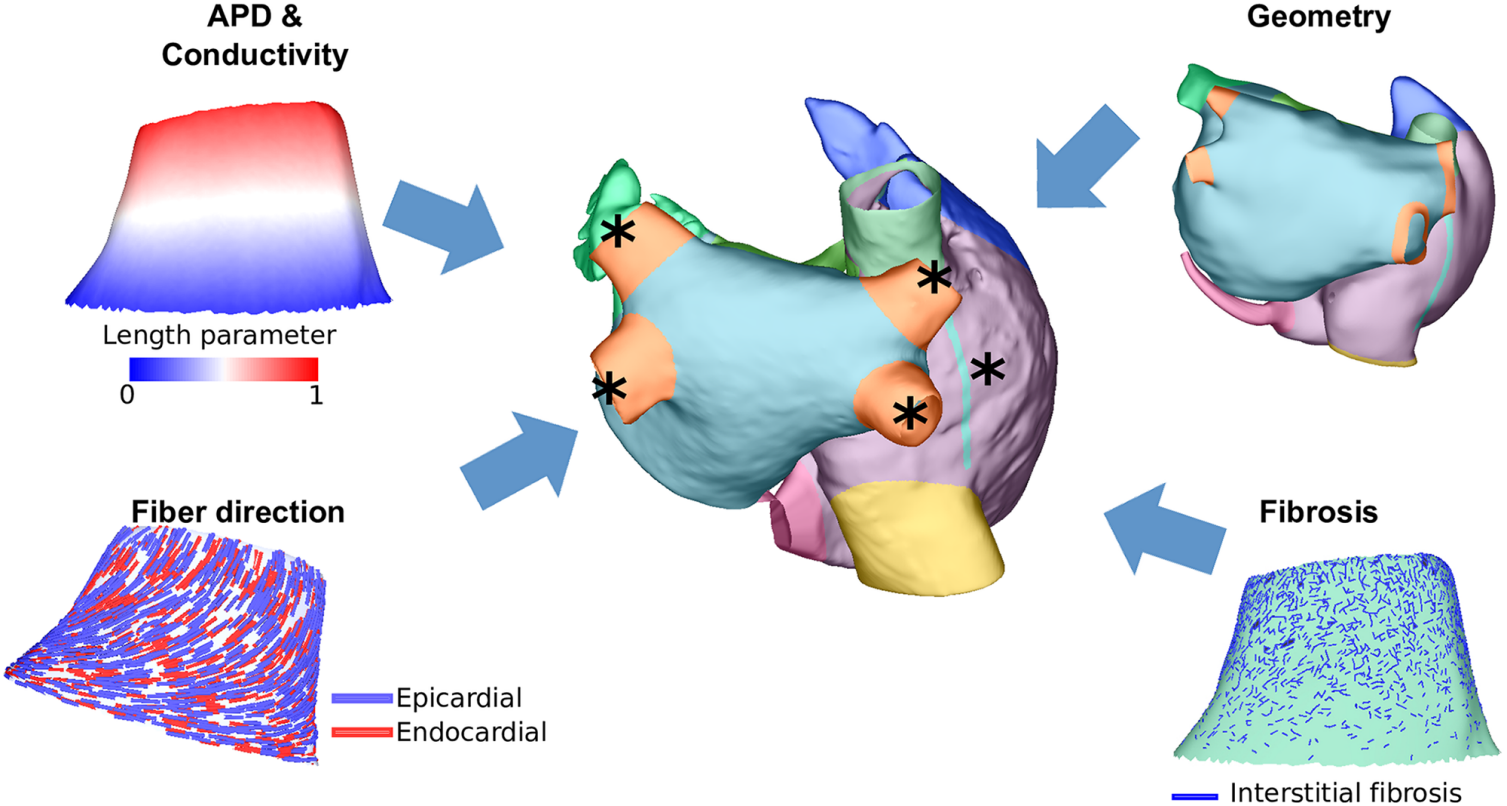
Methods schematic. Schematic showing PV properties varied in this study: APD and conductivity were varied according to a normalized distance parameter indicated by colour; fiber direction (epicardial fibers in blue, endocardial fibers in red); geometry (colours indicate different atrial regions); and, fibrosis (dark blue indicates interstitial fibrosis). Asterisks indicate the pacing locations used for the inducibility pacing protocols.

The following PV properties were varied as shown in schematic Fig 1: APD, CV, fiber direction, the inclusion of fibrosis in the PVs and the atrial geometry. These are described in the following sections.

### Geometry

To investigate the effects of PV length and diameter on arrhythmia inducibility and arrhythmia dynamics, bi-atrial bilayer meshes were constructed from MRI data for twelve patients. All patients gave written informed consent; this study is in accordance with the Declaration of Helsinki, and approved by the Institutional Ethics Committee at the University of Bordeaux. Patient-specific models with electrophysiological heterogeneity and fiber direction were constructed using our modelling pipeline, which uses a universal atrial coordinate system to map scalar and vector data from the original bilayer model to a new patient specific mesh. Late gadolinium enhancement MRI (average resolution 0.625mm x 0.625mm x 2.5mm) was performed using a 1.5T system (Avanto, Siemens Medical Solutions, Erlangen, Germany). These LGE-MRI data were manually segmented using the software MUSIC (Electrophysiology and Heart Modeling Institute, University of Bordeaux, Bordeaux France, and Inria, Sophia Antipolis, France, http://med.inria.fr). The resulting endocardial surfaces were meshed (using the Medical Imaging Registration Toolkit mcubes algorithm [18]) and cut to create open surfaces at the mitral valve, the four pulmonary veins, the tricuspid valve, and each of the superior vena cava, the inferior vena cava and the coronary sinus using ParaView software (Kitware, Clifton Park, NY, USA). The meshes were then remeshed using mmgtools meshing software (http://www.mmgtools.org/), with parameters chosen to produce meshes with an average edge length of 0.34mm to match the resolution of the previously published bilayer model [9]. Two atrial coordinates were defined for each of the LA and RA, which allow automatic transfer of atrial structures to the model, such as the pectinate muscles and Bachmann’s bundle. These coordinates were also used to map fiber directions to the bilayer model.

### PV electrophysiology

To investigate the effects of PV electrophysiology on arrhythmia inducibility and dynamics, we varied PV APD and CV by modifying the value of the inward rectifier current (I_K1_) conductance and tissue level conductivity respectively. I_K1_ conductance was chosen in this case to investigate macroscopic differences in APD [19], although several ionic conductances are known to change with AF [20]. Modifications were either applied homogeneously or following a ostial-distal gradient. This gradient was implemented by calculating geodesic distances from the rim of mesh nodes at the distal PV boundary to all nodes in the PV and from the rim of nodes at the LA/PV junction to all nodes in the PV. The ratio of these two distances was then used as a distance parameter from the LA/PV junction to the distal end of the PV (see Fig 1).

I_K1_ conductance was multiplied by a value in the range 0.5–2.5, resulting in PV APDs in the clinical range of 100–190ms [3, 21, 22]. This rescaling was either a homogeneous change or followed a gradient along the PV length. Gradients of I_K1_ conductance varied from the baseline value at the LA/PV junction, to a maximum scaling factor at the distal boundary. PV APDs are reported at 90% repolarisation for a pacing cycle length of 1000ms. LA APD is 185ms, measured at a LA pacing cycle length of 200ms.

To cover the clinically observed range of PV CVs, longitudinal and transverse tissue conductivities were divided by 1, 2, 3 or 5, resulting in CVs, measured along the PV axis, in the range: 0.28–0.67m/s [3, 21–24]. To model heterogeneous conduction slowing, conductivities were varied as a function of distance from the LA/PV junction, ranging from baseline at the junction to a maximum rescaling (minimum conductivity) at the distal boundary. The direction of this gradient was also reversed to model conduction slowing at the LA/PV junction [5].

### Fibrosis modelling

Motivated by the findings of Hocini et al. [5], interstitial fibrosis was modelled for the PVs with a density varying along the vein, increasing from the LA/PV junction to the distal boundary. This was implemented by randomly selecting edges of elements of the mesh with probability scaled by the distance parameter and the angle of the edge compared to the element fiber direction, where edges in the longitudinal fiber direction were four times more likely to be selected than those in the transverse direction, following our previous methodology [25]. To model microstructural discontinuities, no flux boundary conditions were applied along the connected edge networks, following Costa et al. [26]. An example of modelled PV interstitial fibrosis is shown in S1A Fig.

For a subset of simulations, interstitial fibrosis was incorporated in the biatrial model based on late gadolinium enhancement (LGE)-MRI data, using our previously published methodology [25]. In brief, likelihood of interstitial fibrosis depended on both LGE intensity and the angle of the edge compared to the element fiber direction (see S1B Fig). LGE intensity distributions were either averaged over a population of patients [27], or for an individual patient. The averaged distributions were for patients with paroxysmal AF (averaged over 34 patients), or persistent AF (averaged over 26 patients). For patient-specific simulations, the model arrhythmia dynamics were compared to AF recordings from a commercially available non-invasive ECGi mapping technology (CardioInsight Technologies Inc., Cleveland, OH) for which phase mapping analysis was performed as previously described [28].

### PV fiber direction

PV fiber direction shows significant inter-patient variability. Endocardial and epicardial fiber direction in the four PVs was modified according to fiber arrangements described in the literature [5, 29, 30]. Six arrangements were considered, as follows: 1. circular arrangement on both the endocardium and epicardium; 2. spiralling arrangement on both the endocardium and epicardium; 3. circular arrangement on the endocardium, with longitudinal epicardial fibers; 4. fibers progress from longitudinal at the distal vein to circumferential at the ostium, with identical endocardial and epicardial fibers; 5. epicardial layer fibers as per case 4, with circumferential endocardial fibers; 6. as per case 4, but with a chaotic fiber arrangement at the LA/PV junction. These fiber distributions are shown in S2 Fig.

Cases 4–6 were implemented by setting the fiber angle to be a function of the distance along the vein, measured from the LA/PV junction to the distal boundary, varying from circumferential at the junction to longitudinal at the distal end (representing a change of 90 degrees). The disorder in fiber direction at the LA/PV junction for case 6 was implemented by taking the fibers of case 4 and adding independent standard Gaussian distributions scaled by the distance from the distal boundary, resulting in the largest perturbations at the ostium.

### Pacing protocol to test inducibility

Arrhythmia inducibility was tested by extrastimulus pacing from each of the four PVs individually using a clinically motivated protocol [31], to simulate the occurrence of PV ectopics. Simulations were performed for each of the PVs, to determine the effects of ectopic beat location on inducibility. Sinus rhythm was simulated by stimulating the sinoatrial node region of the model at a cycle length of 700ms throughout the simulation. Each PV was paced individually with five beats at a cycle length of 160ms, and coupling intervals between the first PV beat and a sinus rhythm beat in the range 200–500 ms. Thirty-two pacing protocols were applied for each model set up: eight coupling intervals (coupling interval = 200, 240, 280, 320, 360, 400, 440, 480ms), for each of the four PVs. Inducibility is reported as the proportion of cases resulting in reentry; termed the *inducibility ratio*.

### Pulmonary vein isolation

The effects of PVI were determined for model set-ups that used the original bilayer geometry and in which the arrhythmia lasted for greater than two seconds. PVI was applied two seconds post AF initiation in each case by setting the tissue conductivity close to zero (0.001 S/m) in the regions shown in S3 Fig.

### Model phase singularity analysis

For each case, ten seconds of arrhythmia data were analysed, starting from two seconds post AF initiation, to identify re-entrant waves and wavefront break-up using phase. The phase of the transmembrane voltage was calculated for each node of the mesh using the Hilbert transform, following subtraction of the mean [32]. Phase singularities (PSs) for the transmembrane potential data were identified by calculating the topological charge of each element in the mesh [33], and PS spatial density maps were calculated using previously published methods [14]. PS density maps were then partitioned into the LA body, PV regions, and the RA to assess where drivers were located in relation to the PVs (see S3 Fig). The PV region was defined as the areas enclosed by, and including, the PVI lines; the LA region was then the rest of the LA and left atrial appendage. The *PV PS density ratio* was then defined as the total PV PS count divided by the total model PS count over both atria.

## Results

### PV APD

A difference in APD between the model LA and PVs was required for AF induction. Modelling the PVs using LA cellular properties resulted in non-inducibility, whereas, modelling the LA using PV cellular properties resulted in either non-inducibility or macroreentry.

The effects of modifying PV APD homogeneously or following a gradient are shown in Table 1. Simulations in which PV APD was longer than LA APD were non-inducible (PV APD 191ms). As APD was decreased below the baseline value (181ms), inducibility initially increased and then fluctuated. Comparing cases with equal distal APD, arrhythmia inducibility was significantly higher for APD following a ostial-distal gradient than for homogeneous APD (p = 0.03 from McNemar’s test).

**Table 1:** Inducibility ratios for different PV action potential durations.

Distal APD (ms)	Inducibility (H)	Inducibility (G)
190	0	
181	0.38	
170	0.53	
163	0.38	0.53
152	0.25	
143	0.31	0.41
130	0.53	
120	0.53	0.56
110	0.41	
109	0.41	0.53
100	0.41	

APDs were varied either homogeneously (H) or following a gradient (G). Parameter sets that were not considered are displayed as empty cells.

**Fig 2:**
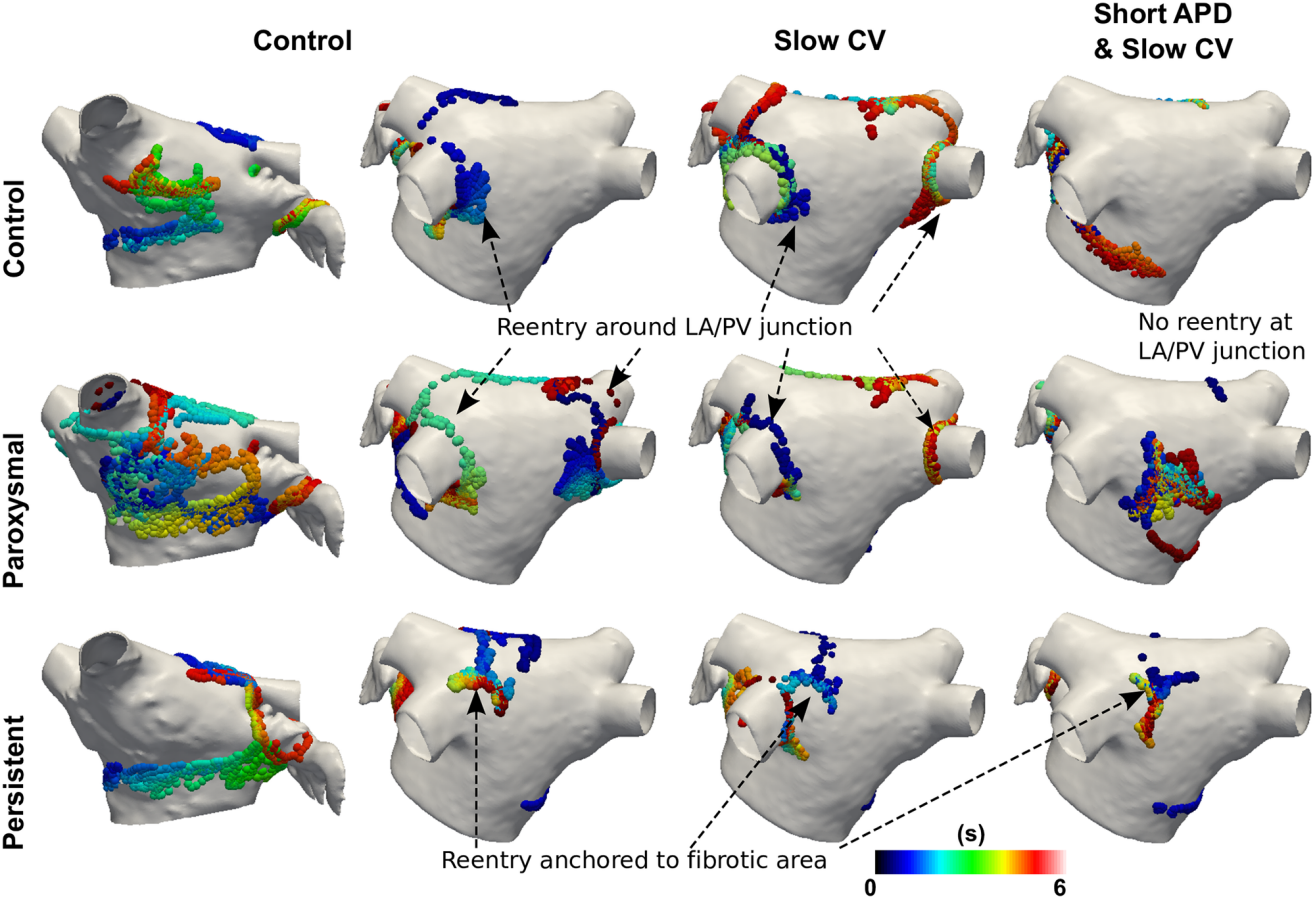
Phase singularity trajectories depend on both PV properties and fibrosis distribution. Trajectories over time for control PV properties (CV: 0.67m/s, APD: 181ms; first two columns, anteroposterior and posteroanterior views); PVs with slow CV (CV: 0.51m/s; middle column); and PVs with short APD and slow CV (CV: 0.51m/s, APD: 120ms; right column). Rows indicate degree of fibrosis, with no fibrosis (control, top row); a distribution averaged over paroxysmal patients (middle row); a distribution averaged over persistent patients (bottom row). Colours indicate the time at which a PS was detected at a site.

PS location was also affected by PV APD. PV PS density was low in cases of short APD, an example of which is shown in Fig 2 where reentry is no longer seen around the LA/PV junction in the case of short APD (120ms). This change was more noticeable for cases with homogeneous PV APD than for a gradient in APD; PV reentry was observed for the baseline case and a heterogeneous APD case, but not for a homogeneous decrease in APD.

### PV CV

Arrhythmia inducibility decreased with homogeneous CV slowing (from 0.38 i.e. 12/32 at 0.67m/s to 0.03 i.e. 1/32 at 0.28m/s). In the baseline model, reentry occurs close to the LA/PV junction due to conduction block when the paced PV beat encounters a change in fiber direction at the base of the PVs, together with a longer LA APD compared to the PV APD. In this case, the wavefront encounters a region of refractory tissue due to the longer APD in the LA. However, when PV CV is slowed homogeneously, the wavefront takes longer to reach the LA tissue, giving the tissue enough time to recover, such that conduction block and reentry no longer occurs. Modifying conductivity following a gradient means that, unlike the homogeneous case, the time taken for the extrastimulus wavefront to reach the LA tissue is similar to the baseline case, so the LA tissue might still be refractory and conduction block might occur. In the case that conduction was slowest at the distal vein, the inducibility was similar to the baseline case (see Table 2, G_*A*_, inducibility is 0.38 at baseline and 0.34 for the cases with CV slowing). Cases with greatest conduction slowing at the LA/PV junction (see Table 2, G_*B*_) exhibit an increase in inducibility (from 0.38 to 0.53) when CV is decreased because of the discontinuity in conductivity at the junction.

**Table 2:** Inducibility for different PV conduction velocities.

CV (m/s)	Inducibility (H)	Inducibility (G_*A*_)	Inducibility (G_*B*_)
0.67	0.38		
0.51	0.41	0.34	0.41
0.34	0.13	0.34	0.38
0.28	0.03	0.34	0.53

CVs were varied either homogeneously (H), or following a gradient with slowest conduction at the distal vein (G_*A*_) or at the LA/PV junction (G_*B*_). Parameter sets that were not considered are displayed as empty cells.

Fig 2 shows that reentry is seen around the LA/PV junction in cases with both baseline and slow CV, indicating that the presence of reentry at the LA/PV junction is independent of PV CV.

### PV fiber direction

PV conduction properties are also affected by PV fiber direction. Modifications in fiber direction increased inducibility compared to the baseline fiber direction (baseline case: 0.38; modified fiber direction cases 1-6: 0.53-0.63). The highest inducibility occurred with circular fibers at the ostium (cases 1 and 4, 0.63), independent of fiber direction at the distal PV end. This inducibility was reduced if the epicardial fibers were not circular at the ostium (case 3, 0.56), or if fibers were spiralling (case 2, 0.56) instead of circular.

### PV properties plus fibrosis

Next we investigated the interplay between PV properties and atrial fibrosis. LA fibrosis properties were varied to represent interstitial fibrosis in paroxysmal and persistent AF patients, incorporating average LGE-MRI distributions [27] into the model. These control, paroxysmal and persistent AF levels of fibrosis were then combined with PV properties varied as follows: baseline CV and APD (0.67m/s, 181ms), slow CV (0.51m/s), short APD (120ms), slow CV and short APD. PS distributions in Fig 2 show that reentry occurred around the LA/PV junction in the case of baseline PV APD for control or paroxysmal levels of fibrosis, but not for shorter PV APD. Modifying PV CV did not affect whether LA/PV reentry is observed. Rotors were found to stabilise to regions of high fibrosis density in the persistent AF case.

**Fig 3:**
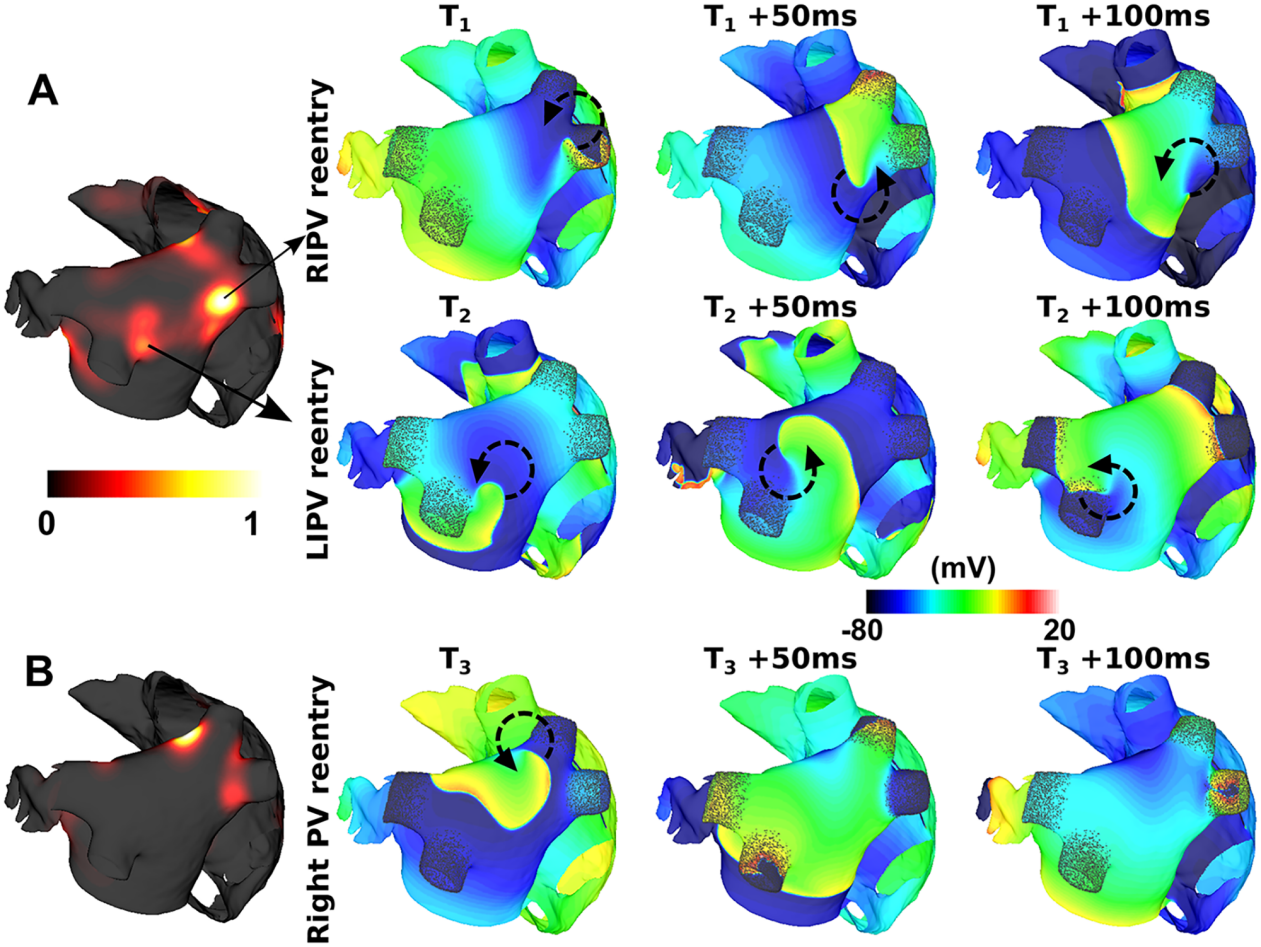
Reentry anchors close to the LA/PV junction in cases with PV fibrosis. The leftmost column shows the normalized phase singularity density map as computed over 10 seconds. The three rightmost columns show snapshots of the transmembrane voltage at three time points. (A) For moderate PV fibrosis, the reentry location changes from the right veins to the left veins, resulting in areas of high PS density close to both sets of veins. (B) For high PV fibrosis, reentry occurs around both of the right PVs. Isopotential maps are at 50ms intervals, with different start times indicted by T_1_, T_2_ and T_3_. Reentry paths are indicated by circular arrows.

Models with PV fibrosis had a higher inducibility compared to the baseline case (0.47 vs. 0.38) and a higher PV PS density since reentry localised there. Fig 3 shows an example with moderate PV fibrosis (A) in which reentry changed from around the RIPV to the LIPV later in the simulation; adding a higher level of PV fibrosis resulted in a more stable reentry around the right PVs (B).

### Patient specific LA fibrosis vs PV properties

**Fig 4:**
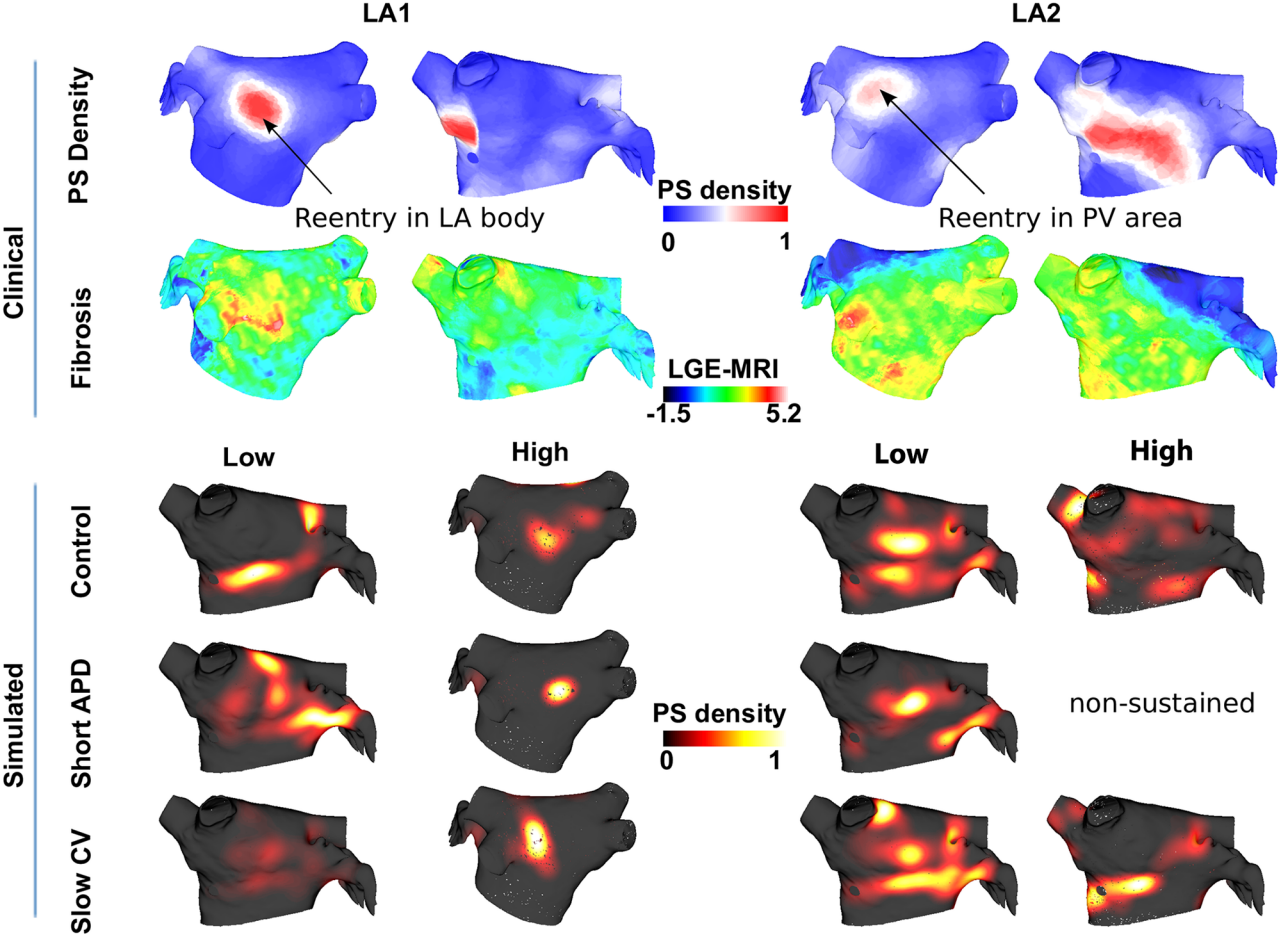
The relative importance of PV properties and atrial fibrosis in determining arrhythmia dynamics varies between patients. Top rows: clinical fibrosis and PS density mapped on to common atria for two patients, shown in posteroanterior and anteroposterior views. Bottom rows: Model PS density maps for low or high levels of fibrosis, and control or short PV APD or slow PV CV. PS densities are displayed in either posteroanterior or anteroposterior view depending on the location of areas of high PS density (LA2 with high fibrosis and short APD was non-sustained).

The relationship between LA fibrosis and PV properties on driver location was investigated on an individual patient basis for four patients. For patients for whom rotors were located away from the PVs (Fig 4 LA1), increasing model fibrosis from low to high increased the model agreement with clinical PS density 2.3 ± 1.0 fold (comparing the sensitivity of identifying clinical regions of high PS density using model PS density between the two simulations). For other patients, lower levels of fibrosis were more appropriate (2.1 fold increase in agreement for lower fibrosis, Fig 4 LA2), and PV isolation converted fibrillation to macroreentry in the model.

### Anatomy

**Fig 5:**
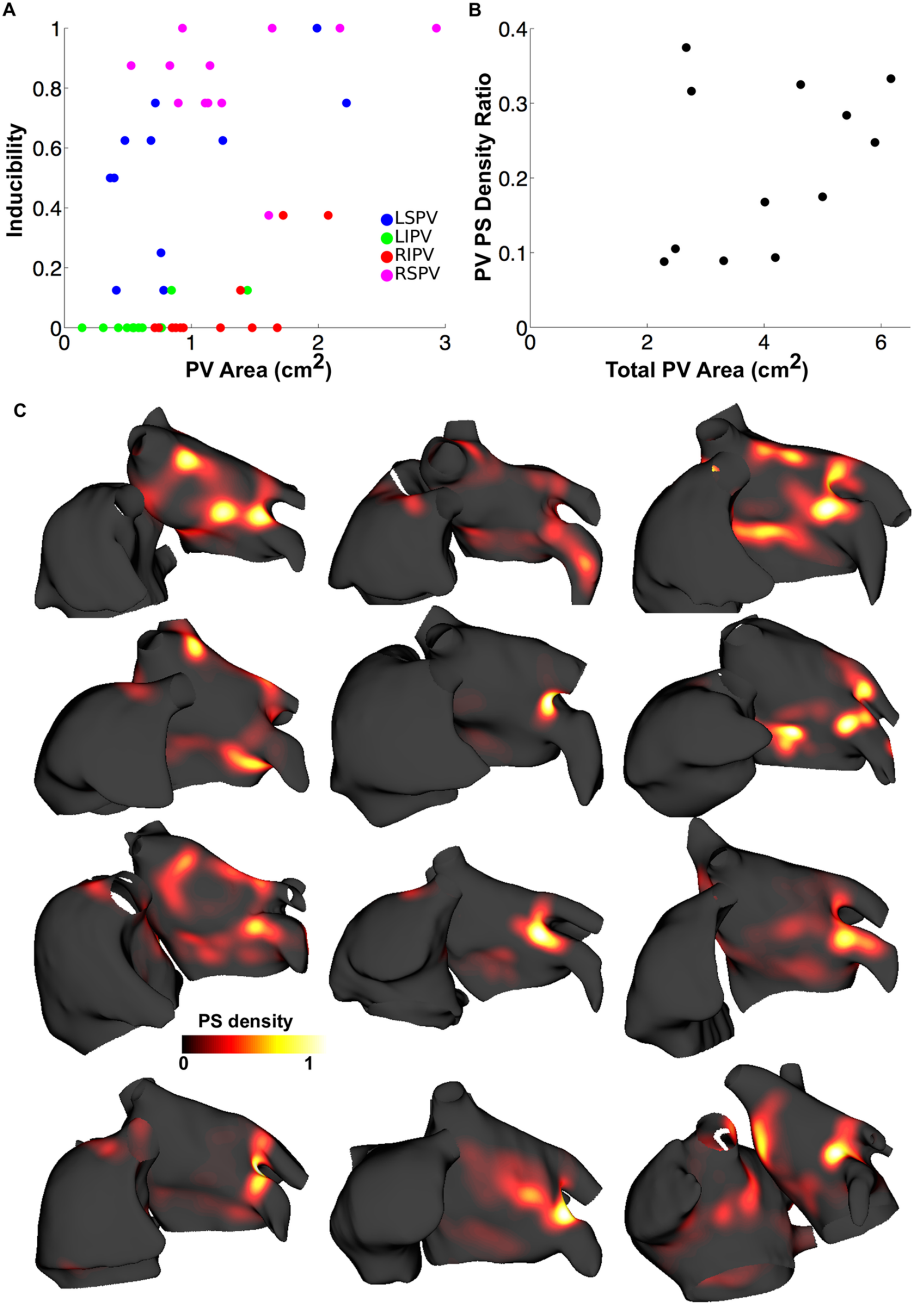
Arrhythmia inducibility and PS density varies between geometries and depends on PV area. (A) Inducibility against PV area for the LSPV (blue), LIPV (green), RIPV (red), RSPV (purple). (B) PV PS density ratio depends on total PV area. (C) Normalised PS density maps for 12 geometries.

Arrhythmia inducibility showed a large variation between patient geometries (0.16–0.47). Increasing PV area increased inducibility to a different degree for each vein: right superior PV (RSPV) inducibility was generally high (> 0.75 for all but one geometry) independent of PV area; left superior PV (LSPV) inducibility increased with PV area (Spearman’s rank correlation coefficient of 0.36 indicating positive correlation; line of best fit gradient 0.27, R^2^ = 0.3); left inferior PV (LIPV) and right inferior PV (RIPV) inducibility exhibited a threshold effect, in which veins were only inducible above a threshold area (Fig 5A). There is no clear relationship between PV length and inducibility. PV PS density ratio increased as PV area increased (Fig 5B, Spearman’s rank correlation coefficient of 0.41 indicating positive correlation). Fig 5C shows that rotor and wavefront trajectories depend on patient geometry, exhibiting varied importance of the PVs compared to other atrial regions.

### Pulmonary vein isolation

**Fig 6:**
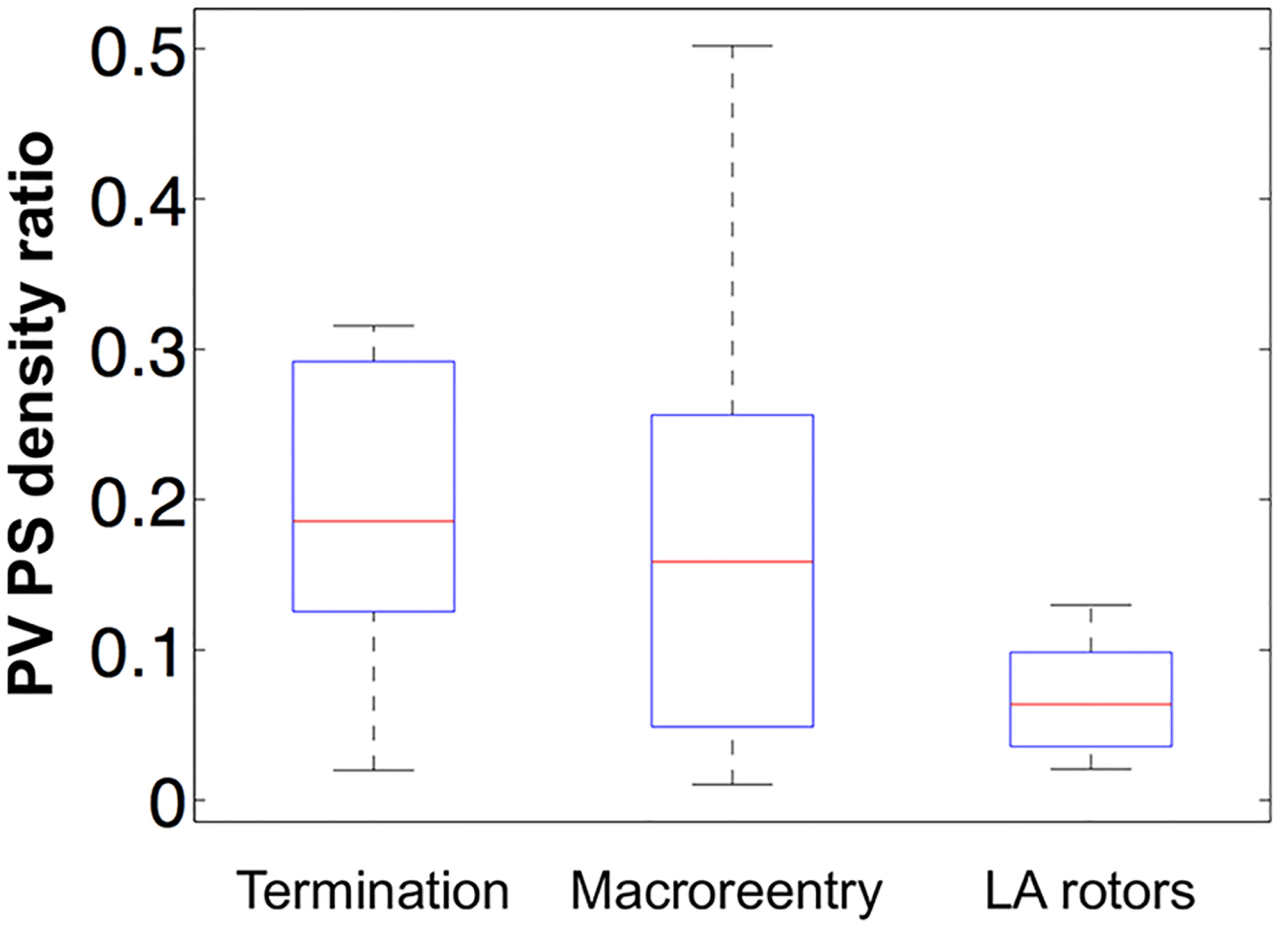
PV PS density ratio is higher for successful PVI cases. Simulation outcomes after PVI were classified as termination (no activity), macroreentry (a large reentry circuit around the LA/PV junctions), or AF sustained by LA rotors (drivers in the LA body). Box plots show PV PS density ratio pre-PVI. Termination PV PS density ratio is significantly higher than for LA rotors (p<0.001), and macroreentry PV PS density ratio is significantly higher than for LA rotors (p = 0.01).

PVI outcome was assessed for cases with varied PV APD (both with a homogeneous change or following a gradient), with the inclusion of PV fibrosis and with varied PV fiber direction because these factors were found to affect the PV PS density ratio. PVI outcome was classified into three classes depending on the activity 1 second after PVI was applied in the model: *termination*, meaning there was no activity; *macroreentry*, meaning that there was a macroreentry around the LA/PV junctions; *AF sustained by LA rotors*, meaning there were drivers in the LA body. These classes accounted for different proportions of the outcomes: termination (27.3% of cases), macroreentry (39.4%), or AF sustained by LA rotors (33.3%). Calculating the PV PS density ratio before PVI for each of these classes shows that cases in which the arrhythmia either terminated or changed to a macroreentry are characterised by a statistically higher PV PS density ratio pre-PVI than cases sustained by LA rotors post-PVI (see Fig 6, t-test comparing termination and LA rotors shows they are significantly different, p<0.001; comparing macroreentry and LA rotors p = 0.01). High PV PS density ratio may indicate likelihood of PVI success.

## Discussion

### Main findings

In this computational modelling study, we demonstrated that the PVs can play a large role in arrhythmia maintenance and initiation, beyond being simply sources of ectopic beats. We separated the effects of PV properties and atrial fibrosis on arrhythmia inducibility, maintenance mechanisms and the outcome of PVI, based on population or individual patient data. PV properties affect arrhythmia susceptibility from ectopic beats; short PV APD increased arrhythmia susceptibility, while longer PV APD was found to be protective. Arrhythmia inducibility increased with slower CV at the LA/PV junction, but not for cases with homogeneous CV changes or slower CV at the distal PV. The effectiveness of PVI is usually attributed to PV ectopy, but our study demonstrates that the PVs affect reentry in other ways and this may, in part, also account for success or failure of PVI. Both PV properties and fibrosis distribution affect arrhythmia dynamics, which varies from meandering rotors to PV reentry (in cases with baseline or long APD), and then to stable rotors at regions of high fibrosis density. PS density in the PV region was high for cases with PV fibrosis. The measurement of fibrosis and PV properties may indicate patient specific susceptibility to AF initiation and maintenance. PV PS density before PVI was higher in cases in which AF terminated or converted to a macroreentry; thus, high PV PS density may indicate likelihood of AF termination by PVI alone.

### PV APD

PV repolarisation is heterogeneous in the PVs [23], and exhibits distinct properties in AF patients, with Rostock et al. reporting a greater decrease in PV ERP than LA ERP in patients with AF, termed AF begets AF in the PVs [21]. Jais et al. found that PV ERP is greater than LA ERP in AF patients, but this gradient is reversed in AF patients [3]. ERP measured at the distal PV is shorter than at the LA/PV junction during AF [5, 22]. Motivated by these clinical and experimental studies, we modelled a decrease in PV APD, which was applied either homogeneously, or as a gradient of decreasing APD along the length of the PV, with the shortest APD at the distal PV rim. An initial decrease in APD increased inducibility (Table 1), which agrees with clinical findings of increased inducibility for AF patients. Applying this change following a gradient, as observed in previous studies, led to an increased inducibility compared to a homogeneous change in APD. Similar to Calvo et al. [34] we found that rotor location depends on PV APD (Fig 2). Thus PV APD affects PVI outcome in two ways; on the one hand, decreasing APD increases inducibility, emphasising the importance of PVI in the case of ectopic beats; on the other hand, PV PS density decreases for cases with short PV APD, and PVI was less likely to terminate AF.

### PV CV

Multiple studies have measured conduction slowing in the PVs [3, 5, 21–24]. We modelled changes in tissue conductivity either homogeneously, or as a function of distance along the PV. Simply decreasing conductivity and thus decreasing CV, decreased inducibility (Table 2). Kumagai et al. reported that conduction delay was longer for the distal to ostial direction [22]. We found that modifying conductivity following a gradient, with CV decreasing towards the LA/PV junction, resulted in an increase in inducibility in the model. This agrees with the clinical observations of Pascale et al. [1]. This suggests that PVI should be performed in cases in which CV decreases towards the LA/PV junction as these cases have high inducibility. Changes in CV may also be due to other factors, including gap junction remodelling, modified sodium conductance or changes in fiber direction [5, 29].

### PV fiber direction

A variety of PV fiber patterns have been described in the literature and there is variability between patients. Interestingly, all of the PV fiber directions considered in our study showed an increased inducibility compared to the baseline model. Verheule et al. [29] documented circumferential strands that spiral around the lumen of the veins, motivating the arrangements for cases 1 and 4 in our study; Aslanidi et al. [15] reported that fibers run in a spiralling arrangement (case 2); Ho et al. [30] measured mainly circular or spiral bundles, with longitudinal bundles (cases 3 and 5); Hocini et al. [5] reported longitudinal fibers at the distal PV, with circumferential and a mixed chaotic fiber direction at the PV ostium (case 6). Using current imaging technologies, PV fiber direction cannot be reliably measured in vivo.

In our study, fiber direction at the PV ostium was found to be more important than at the distal PV; the greatest inducibility was for cases with circular fibers at the ostium on both endocardial and epicardial surfaces, independent of fiber direction at the distal PV end. Similar to modelling studies by both Coleman [35] and Aslanidi [15], inducibility increased due to conduction block near the PVs.

### PV anatomy

PVs may be larger in AF patients compared to controls [4, 36], and this difference may vary between veins; Lin et al. found dilatation of the superior PVs in patients with focal AF originating from the PVs, but no difference in the dimensions of inferior PVs compared to control or to patients with focal AF from the superior vena cava or crista terminalis [37]. We found that inducibility increased with PV area for the LSPV, LIPV and RIPV, but not for the RSPV (see Fig 5). In addition, PV PS density ratio increased with total PV area, suggesting that PVI alone is more likely to be a successful treatment strategy in the case of larger veins. However, Den Uijl et al. found no relation between PV dimensions and the outcome of PVI [38]. Rotors were commonly found in areas of high surface curvature, including the LA/PV junction and left atrial appendage ostia, which agrees with findings of Tzortzis et al. [39]. However, there were differences in PS density between geometries, with varying importance of the LA/PV junction (Fig 5), demonstrating the importance of modelling the geometry of an individual patient.

### Atrial fibrosis

Myocardial tissue within the PVs is significantly fibrotic, which may lead to slow conduction and reentry [5, 30, 40]. More fibrosis is found in the distal PV, with increased connective tissue deposition between myocardial cells [41]. We modelled interstitial PV fibrosis with increasing density distally, and found that the inclusion of PV fibrosis increased PS density in the PV region of the model due to increased reentry around the LA/PV junction and wave break in the areas of fibrosis. This, together with the results in Fig 6, suggests that PVI alone is more likely to be a successful in cases of high PV fibrosis. There are multiple methodologies for modelling atrial fibrosis [25, 42, 43], and the choice of method may affect this localisation.

Population based distributions of atrial fibrosis were modelled for paroxysmal and persistent patients, together with varied PV properties. The presence of LA/PV reentry depends on both PV properties and the presence of fibrosis; reentry is seen at the LA/PV junction for cases with baseline PV APD, but not for short PV APD, and stabilised to areas of high fibrosis in persistent AF, for which LA/PV reentry no longer occurred. This suggests that rotor location depends on both fibrosis and PV properties. This finding may explain the clinical findings of Lim et al. in which drivers are primarily located in the PV region in early AF, but AF complexity increased with increased AF duration, and drivers are also located at sites away from the PVs [6]. During early AF, PV properties may be more important, while with increasing AF duration, there is increased atrial fibrosis in the atrial body that affects driver location. This suggests that in cases with increased atrial fibrosis in the atrial body, ablation in addition to PVI is likely to be required.

Simulations of models with patient-specific atrial fibrosis together with varied PV properties performed in this study offer a proof of concept for using this approach in future studies. The level of atrial fibrosis and PV properties that gave the best fit of the model PS density to the clinical PS density varied between patients. Measurement of PV ERP and conduction properties using a lasso catheter before PVI could be used to tune the model properties, together with LGE-MRI or an electro-anatomic voltage map.

### Ablation strategies

It is difficult to predict whether PVI alone is likely to be a successful treatment strategy for a patient with persistent AF [44]. This will depend on both the susceptibility to AF from ectopic beats, together with electrical driver location, and electrical size. Our study describes multiple factors that affect the susceptibility to AF from ectopic beats. Measurement of PV APD, PV CV and PV size will allow prediction of the susceptibility to AF from ectopic beats. Arrhythmia susceptibility increased in cases with short PV APD, slower CV at the LA/PV junction and larger veins, suggesting the importance of PVI in these cases.

The likelihood that PVI terminates AF was also found to depend on driver location, assessed using PS density. Our simulation studies suggest that high PV PS density indicates likelihood of PVI success. Thus either measuring this clinically using non-invasive ECGi recordings, or running patient-specific simulations to estimate this value may suggest whether ablation in addition to PVI should be performed. In a recent clinical study, Navara et al. observed AF termination during ablation near the PVs, before complete isolation, in cases where rotational and focal activity were identified close to these ablation sites [45]. These data may support the PV PS density metric suggested in our study. Our simulations show that PV PS density depends on PV APD, the degree of PV fibrosis and to a lesser extent on PV fiber direction. To the best of the authors’ knowledge, there are no previous studies on the relationship between fibrosis in the PVs, or PV fiber direction, and the success rate of PVI. Measuring atrial electrogram properties, including AF cycle length, before and after ablation may indicate changes in local tissue refractoriness [46]. PV APD can be estimated clinically by pacing to find the PV ERP; and PV fibrosis may be estimated using LGE-MRI, although this is challenging, as the tissue is thin. PV fiber direction data is not currently available clinically, which limits the predictive ability of the model. Areas of high PV PS density on ECGi need to be carefully interpreted in terms of expected accuracy of the inverse solution on the PVs and the incidence of false phase singularity detection [47]. In addition, multiple mechanisms may underlie areas of high PS density. Importantly, not all PSs sustain and drive AF, and represent suitable targets for ablation.

### Limitations

Limitations to this study include that PV branching structures were not considered since PVs were trimmed at the highest level that results in a single PV rim at each distal PV. Mansour et al. found that just 56% of patients had four PVs with separate ostia [48], 29% of patients had an additional PV, and 17% a common PV trunk. Although some studies have reported differences in ERP between the endocardium and epicardium [23], we modelled the endocardium and epicardium ERP identically. Furthermore, we modelled changes in APD by modifying I_K1_ only and did not consider other ionic conductances or methods for parametrisation [20, 49, 50]. We used a bilayer model, rather than a volumetric model incorporating thickness, which will affect rotor drift [51]. In addition, we did not model changes in connexins [29] or cell morphology [52]. Furthermore, we modelled 2 seconds of activity following PVI in the model, where these ablation lesions were applied simultaneously rather than sequentially as in the clinic, and we did not model long term AF recurrence. Finally, we did not consider the case of AF sustained by focal beats; we either considered the inducibility due to PV ectopics, or maintenance due to reentry.

### Conclusion

Our computational modelling study suggests that measurement of fibrosis and PV properties may indicate patient specific susceptibility to AF initiation and maintenance. In addition, high PV PS density pre-ablation indicates likelihood of PVI success in our simulations, motivating a retrospective clinical study into this metric.

## Supporting information

S1 Fig Modelling interstitial fibrosis. (A) PV interstitial fibrosis is included with probability depending on the fiber direction and length along the PV. (B) Interstitial fibrosis is modelled with probability scaled by the LGE intensity.
(TIF)

S2 Fig PV fiber directions. Fiber directions for the epicardium in blue and endocardium in red for the baseline model and six other cases.
(TIF)

S3 Fig PVI lines and regions used for PS density analysis. Left: PVI lines shown in blue; Right: regions for PS density analysis (red: PVs, black: LA, yellow: RA).
(TIF)
