# Supplementary figures and images for: Variability in pulmonary vein electrophysiology and fibrosis determines arrhythmia susceptibility and dynamics
Source: PLoS Comput Biol. 2018 May 24;14(5):e1006166. doi: 10.1371/journal.pcbi.1006166 (PMC5997352; doi:10.1371/journal.pcbi.1006166)

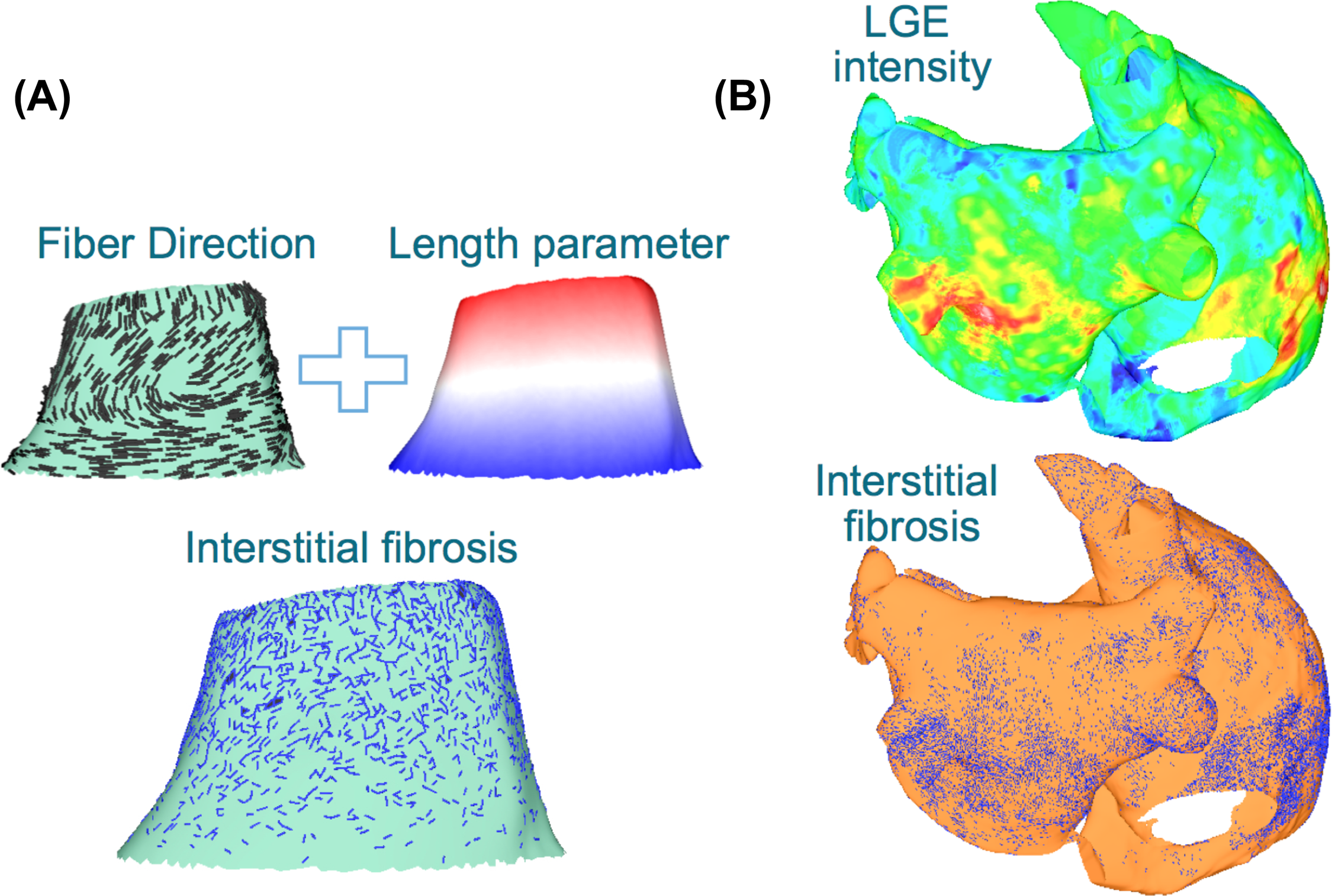

Supplement: S1 Fig — (A) PV interstitial fibrosis is included with probability depending on the fiber direction and length along the PV. (B) Interstitial fibrosis is modelled with probability scaled by the LGE intensity. (TIF) [file pcbi.1006166.s001.tif]

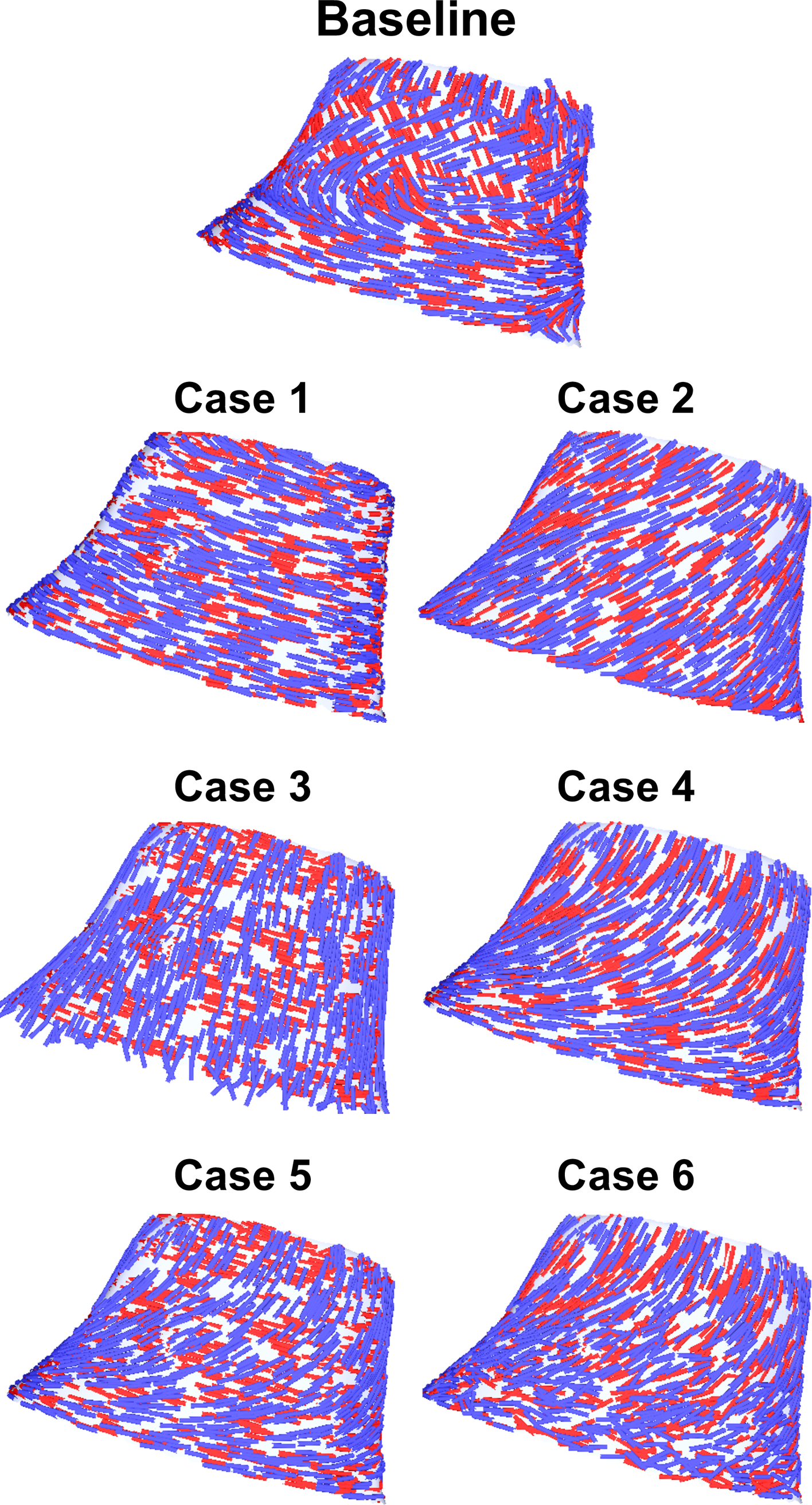

Supplement: S2 Fig — Fiber directions for the epicardium in blue and endocardium in red for the baseline model and six other cases. (TIF) [file pcbi.1006166.s002.tif]

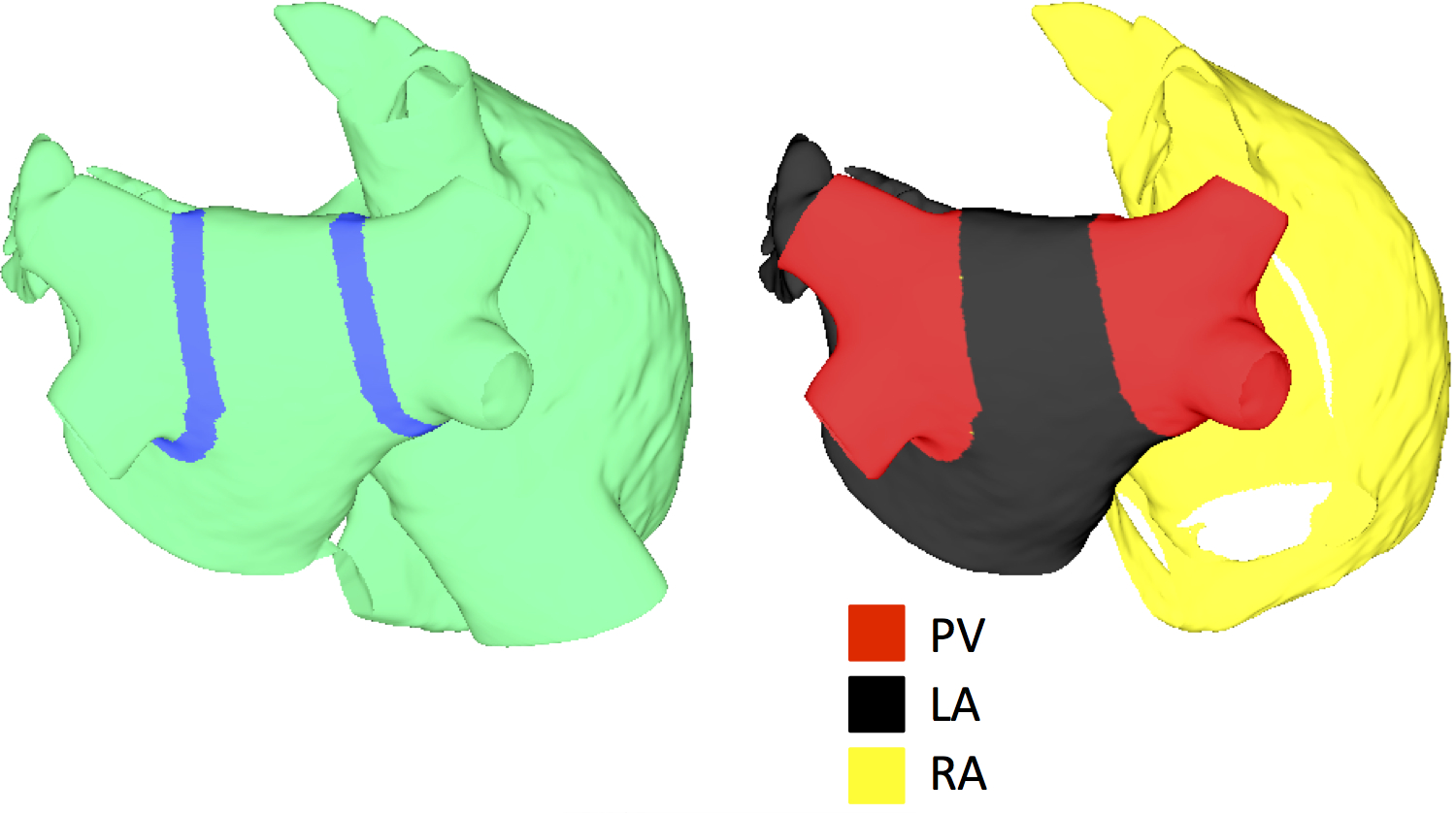

Supplement: S3 Fig — Left: PVI lines shown in blue; Right: regions for PS density analysis (red: PVs, black: LA, yellow: RA). (TIF) [file pcbi.1006166.s003.tif]
